# A Rare Etiology of Abnormally Large Gastric Folds: Menetrier's Disease

**DOI:** 10.1155/2019/7927083

**Published:** 2019-12-30

**Authors:** Muhammad Umar Kamal, Hassan Tariq, Vaniza Mehak, Sara Azam, Kishore Kumar, Masooma Niazi, Anil Dev

**Affiliations:** ^1^Department of Medicine, BronxCare Health System, Bronx, NY 10457, USA; ^2^Division of Gastroenterology, Department of Medicine, BronxCare Health System, Bronx, NY 10457, USA; ^3^Department of Pathology, BronxCare Health System, Bronx, NY 10457, USA

## Abstract

Menetrier's disease (MD) is described as hypertrophied giant gastric folds causing hypoproteinemia due to protein losing gastropathy and is associated with higher risk of gastric adenocarcinoma. We present a case of a 58-year-old male who presented to our clinic with Melena and endoscopic work up showed enlarged gastric folds and erythematous mucosa in the antrum and three nonbleeding angioectasias in the duodenum. Mucosa biopsies were negative for *H. pylori* infection. He underwent polypectomy which showed fundic gland polyps. After 1½ years, EGD was repeated for abnormal computerized tomography of abdomen which showed enlarged gastric folds and biopsy revealed gastric fundic mucosa with foveolar hyperplasia, dilated fundic glands, and chronic gastritis. Stomach biopsy results were consistent with MD. Our patient had progressive disease in one and half years. It is important to follow patient with large gastric folds regular as they can develop MD over time which has increased risk of gastric adenocarcinoma.

## 1. Introduction

MD is a rare clinical disease which is characterized by abnormal hypertrophy of gastric folds; causing hypoproteinemia due to protein losing gastropathy [1]. It also causes hypochloremia and associated with increased chances of gastric adenocarcinoma. The pathophysiology is not exactly clear, but it is hypothesized that up regulation of Transforming growth factor (TGF) is the underlying mechanism of cellular changes in gastric mucosa [2]. It is also noted that enormous secretions of thick mucus and transforming growth factor alpha (TGF-*α*) contribute towards gastric hypertrophy [3]. Some authors have discussed its linkage to *Helicobacter pylori* (*H. pylori*) infection [4].

The disease commonly affects the middle-aged males but younger pediatric population is also involved [5, 6]. The clinical features include upper abdominal pain and discomfort, nausea, vomiting, weight loss, and swelling of the body due to hypoalbumemia. Severe disease lead to intractable protein loss, upper gastrointestinal bleeding or gastric cancers [7].

Endoscopically giant gastric folds are visualized and on microscopic examination hyperplasia of the mucus-secreting surface cells is seen commonly in the gastric pits which is called foveolar epithelium. The hypertrophy of the foveolar epithelium results in replacement of the normal gastric glandular structure. These changes mainly occur in the gastric body where gastric glands containing chief cells are located [4].

The disease is diagnosed with clinical features and further laboratory testing including serum albumin levels, EGD, and testing for *H. pylori* [4, 7].

It is commonly treated with proton pump inhibitors, high protein diet, *H. pylori* eradication, cetuximab (monoclonal antibody), and somatostatin analog injections octreotide long-acting release [4, 6, 8]. In severe progressive disease and medically resistant cases, total and partial gastrectomy are required for management.

## 2. Case Presentation

A 58-year-old male with comorbidities of hypertension, chronic obstructive pulmonary disease, benign prostatic hyperplasia, and gastro-esophageal reflux disease was initially referred to the gastroenterology clinic by primary care physician for the surveillance colonoscopy, and evaluation of dark colored stools.

Patient noted dark colored stools during the Christmas weekend associated with nausea, vomiting, and abdominal pain. The abdominal pain was epigastric, gradual onset, severe in intensity, nonradiating, aggravated with emesis with no specific relieving factors. Denies any hematemesis. He had colonoscopy 10 years ago and had history of colonic polyps. At the time of assessment in the clinic he denied any nausea, vomiting, and worsening abdominal pain or other symptoms.

Physical examination of cardiovascular, respiratory, gastrointestinal, and neurological system was unremarkable. Vitals were within normal limits. Recent laboratory workup showed hemoglobin of 14.7 g/dl with normal renal and hepatic parameters including normal albumin levels. He has past medical history of anemia and esophagogastroduodenoscopy (EGD) and Colonoscopy which revealed arteriovenous malformations (AVMs). He also has history of Alcoholic pancreatitis, alcohol dependence, smoking but no history of any drug abuse. Family history was negative for the colon cancer and stomach cancer. For gastroesophageal reflux disease, he was prescribed PPIs as needed but he was not taking it regularly due to little or no relief from them.

For the evaluation of GI bleed, he underwent colonoscopy in January 2016 which showed normal ileum, few 2–4 mm hyperplastic polyps in the sigmoid colon, and few 3 mm polyps in the rectum which had reactive lymphoid aggregate and focal epithelial hyperplasia on biopsy. Other findings included diverticulosis in the sigmoid colon and in the descending colon and nonbleeding internal hemorrhoids. In January 2016 an EGD showed normal esophagus but enlarged gastric folds and erythematous mucosa in the antrum and stomach biopsy showed gastric antral mucosa with focal mild increase of eosinophils and congestion. Three nonbleeding angioectasias were seen in the duodenum which were treated with argon plasma coagulation (APC). Mucosa biopsies were negative for *H. pylori* infection. For evaluation of the AVMs, Small bowel enteroscopy was done on February 2016 which showed mucosal changes in the jejunum and biopsy revealed small bowel mucosa with dilated lymphatics. EGD was repeated for polypectomy in February 2016 which showed enlarged gastric folds and were ligated. There was normal duodenal bulb and mucosal resection was successfully performed. The biopsy was consistent with fundic gland polyps.

**Figure 1:**
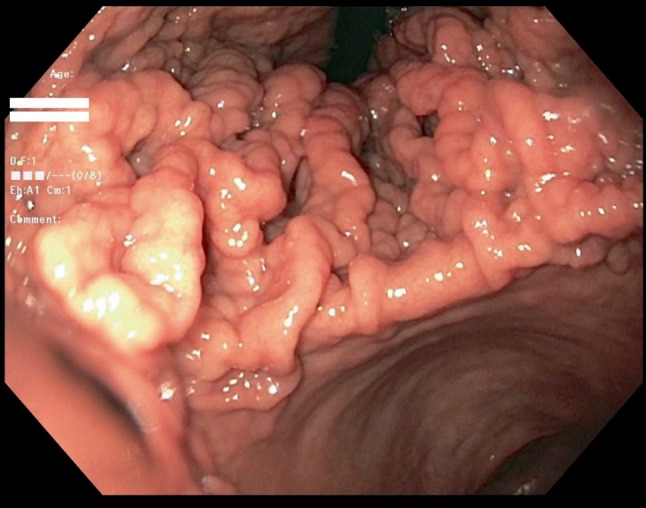
Esophagogastroduodenoscopy showing diffusely thick gastric folds were found in the gastric fundus and in the gastric body with characteristic sparing of antrum.

**Figure 2:**
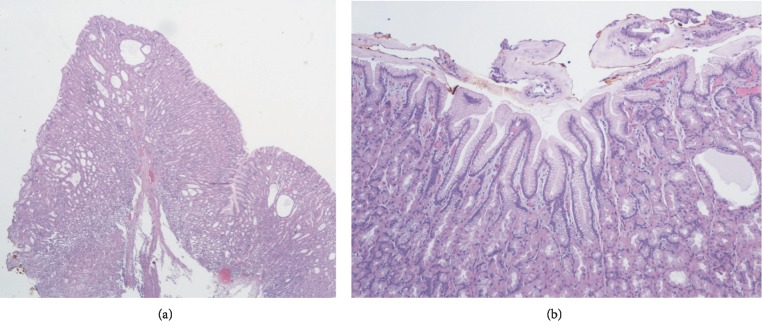
(a) Gastric folds biopsy on low power magnification showing mucosal thickening with foveolar hyperplasia and cystic dilatation of the gland (H and E, magnification ×2) consistent with Menetrier disease. (b) Another section of the gastric folds biopsy showing foveolar hyperplasia with excessive mucous spills on the luminal surface and a cystically dilated gland (H and E, magnification ×100).

He was referred to the gastroenterology clinic after eighteen months for abnormal abdominal computerized tomography findings of gastric wall thickening which he had for abdominal pain. This epigastric pain was gradual in onset, severe in intensity, radiating to right upper quadrant, and progressively worsened. To rule out gastric malignancy, EGD and EUS were done. EGD in September 2017 showed giant gastric folds, much larger than seen on the prior endoscopies. This is shown in Figure 1. The biopsied enlarged gastric folds revealed gastric fundic mucosa with foveolar hyperplasia, dilated fundic glands and chronic gastritis which is shown in Figure 2. Another gastric fundus nodule biopsy revealed gastric fundic mucosa with foveolar hyperplasia and mild chronic inflammation.

**Figure 3:**
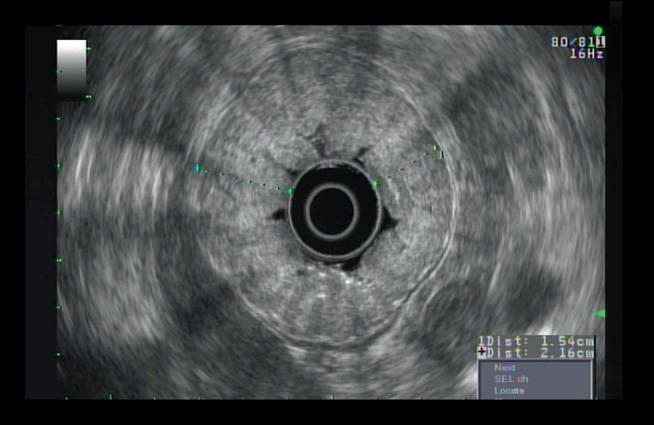
Endoscopic ultrasound showing wall thickening was seen in the body of the stomach. The thickening appeared to be primarily within the sub mucosa (Layer 3), >1 cm.

With the background of thickened gastric folds and episodes of recurrent pancreatitis, the patient underwent upper endoscopy ultrasound for the evaluation of the pancreas as well as the stomach on March 2018 which showed wall thickening in the body of the stomach. The thickening appeared to be primarily within the submucosa (Layer 3) of the stomach (Figure 3). There was no sign of significant pathology in the common bile duct, gall bladder, and the main pancreatic duct.

Stomach biopsy results were consistent with Menetrier disease. Patient was followed in the clinic and explained the risks of adenocarcinoma associated with this disease. Our patient has worsening of the disease in one and half years.

## 3. Discussion

MD is a diagnostic challenge due to the rare occurrence of the disease, complex pathophysiology and the lack of precise diagnostic criteria [7, 9]. Our case is a unique example of MD that is without hypoalbuminemia and without gastric colonization with *H. pylori* like other similar cases reported in literature [5, 10]. This points out to the fact that further research is needed in understanding the pathophysiology of the disease in addition to *H. pylori* infection and low serum albumin levels.

It is well known that adult MD occurs in the setting of *H. pylori* infection of the stomach [11, 12]. In 1993, Bayerdorffer et al. evaluated about 138 cases of hypertrophic gastropathy and found more than 90% of patient having biopsies positive for *H. pylori* [13]. It is hypothesized cyclooxygenase (COX)-2 induced by *H. pylori* infections can result in antiapoptotic effect on the mucus secreting gastric cells resulting in hypertrophy of the glandular epithelium causing excessive protein and chloride secretion [14].

Although there are case reports of MD in the absence of *H. pylori* infection in the biopsied gastric tissues. This suggests that *H. pylori* may not be the causative agent but possibly a contributor towards the development of foveolar hyperplasia and gastric hypertrophy [14]. Low albumin level is an important finding for the diagnosis of MD [15] but in literature normal albumin levels in MD are mentioned in adults and children [5, 6, 10]. In our case, the serum albumin level of the patient was 4.1 g/dl without associated edema and never reduced during the follow up clinical visits. This speculates that our patient might have a new subtype of MD.

The pathophysiology of MD is explained by the upregulated signaling of epidermal growth factor receptor (EGFR) which is the effect of increased production of TGF-*α*). This finding is based on transgenic mice with MD who were found to have elevated TGF-*α* levels in gastric mucosa. Endo et al. also found high levels of TGF-*α* in the hypertrophied foveolar gastric mucosa. These stimulate EGFR which are usually found mainly in the parietal cells are expressed on the lumen side of foveolar cells [3, 14]. Ligand binding to EGFR results in a cascade of proliferative events; ultimately accounting for hypertrophic gastropathy. The inducers of the TGF-*α*/EGFR signaling pathway are not well known but certain infectious agents are mentioned in literature. This include cytomegalovirus (CMV) and *H. pylori*. Wang et al. reported that CMV proteins stimulate the pathway of hypertrophy and proliferation [16]. This pathway is common in but rarely present in adults except in immunocompromised states [17]. However, in our case *H. pylori* was not found. Different possible etiologies of the large gastric fold as mentioned in Figure 1.

This disease commonly manifests with epigastric pain and low albumin levels due to protein losing gastropathy [18]. Other symptoms include nausea, vomiting, diarrhea, weight loss, anorexia, early satiety, gastrointestinal bleed, fatigue, and edema [5]. The disease is progressive and affects men more than women with average age of diagnosis of 55 years [3]. The disease typically involves the whole fundus, body, and upper part of the gastric mucosa with sparing of the antrum [19]. However, there are case reports of involvement of the antrum [20].

MD mimics a wide variety of other diseases. These consist of different forms of polyps and polyposis syndromes like hyperplastic polyps or juvenile polyps or juvenile polyposis syndrome. These are differentiated from MD with detailed history including family history, additional clinical manifestations, endoscopic imaging, and histopathological analysis and genetic analysis. PPI can also cause hyperplasia of the gastric mucosa and mimics MD but in these cases histological examinations reveal predominance of parietal cells along with occasional dilation of the oxyntic glands. In sporadic cases, the apex of the parietal cell snout within the lumen of the gastric glands [21]. These findings were not seen in our histological examination which differentiate PPI induced hyperplastic glands from MD. In addition, gastrin producing tumors like Zollinger–Ellison syndrome also shows parietal cell hyperplasia in histopathological examination. MD in contrast has decreased appearance of parietal cell mass. It is essential to rule out other differentials of thickened gastric walls like malignancies (lymphomas, gastric cancers, gastrointestinal stromal tumors), infections (tuberculosis), or other infiltrative diseases [22].

Management of MD includes *H. pylori* eradication, use of octreotide for the modulation of the TGF-*α*/EGFR signaling, antibody binding and inhibiting the EGFR or surgical treatments. Many studies have mentioned positive results with *H. pylori* eradication using proton pump inhibitors and antibiotics [12, 23]. However, in *H. pylori* negative cases it is assumed that the disease will not respond to the eradication regimen of *H. pylori*. Some evidence suggests the somatostatin analogs like octreotide help in relieving symptoms but effect on the disease progression and development of malignancies is not clear [24, 25]. It is found that chronic cases of MD respond well to the monoclonal AB-cetuximab [8]. But cases refractory to the medical therapy, progressively worsening disease and for prevention of gastric cancer, gastrectomy is the best option. Partial gastrectomy is done with the use of laparoscopic technique [26]. Total gastrectomy is preferred despite MD sparing the antrum as risk of leakage from the anastomotic inflamed tissue is very high [7, 10]. However, there are cases of successful limited gastric resection in cases of MD localized to the gastric antrum [5]. This technique needs further exploration in these cases. Also, the percentage of risk of gastric adenocarcinoma with limited gastrectomy need to be further determined.

It is very important to regularly follow up cases of MD as there is high risk of gastric tumors likely adenocarcinomas and lymphomas [15, 27, 28]. MD is considered as a premalignant condition and it reasonable to have regular follow up endoscopies to monitor the developed for cancers in this precancerous state. Cases are mentioned in literature where surveillance endoscopies were done 1-2 years to monitor the MD [15]. There is no definite time internal for the developed of the cancer in MD. In literature, cases are mentioned of simultaneous occurrence of MD and gastric malignancy [29, 30]. Based on the limited data of case reports, the cancer developments were mentioned from 3.5 to 16 years since the initial diagnosis of MD [15, 28, 31]. Therefore, it is reasonable to monitor malignant transformation of MD for enough time. Patient should be notified about the possibility of cancer in MD and patient who are very concerned may opt to definitive treatment in the form of partial or total gastrectomy [15].

## 4. Conclusion

This disease should be considered in patients coming with upper abdominal symptoms and endoscopic findings of large abnormal gastric folds with or without evidence of *H. pylori* and hypoalbuminemia.

Polypectomy is essential for the diagnosis and patient can be managed medically initially. Surgical treatment is considered in cases refractory to medical management or in patients with suspected gastric malignancy or obstructive symptoms. As there is high evidence of malignant transformations in MD, regular endoscopic surveillance of the gastric mucosa is required.
